# The impact of *PICALM* genetic variations on reserve capacity of posterior cingulate in AD continuum

**DOI:** 10.1038/srep24480

**Published:** 2016-04-27

**Authors:** Wei Xu, Hui-Fu Wang, Lin Tan, Meng-Shan Tan, Chen-Chen Tan, Xi-Chen Zhu, Dan Miao, Wan-Jiang Yu, Teng Jiang, Lan Tan, Jin-Tai Yu, Alzheimer’s Disease Neuroimaging Initiative Group, Michael W. Weiner, Paul Aisen, Ronald Petersen, Clifford R. Jack, William Jagust, John Q. Trojanowki, Arthur W. Toga, Laurel Beckett, Robert C. Green, Andrew J. Saykin, John Morris, Leslie M. Shaw, Jeffrey Kaye, Joseph Quinn, Lisa Silbert, Betty Lind, Raina Carter, Sara Dolen, Lon S. Schneider, Sonia Pawluczyk, Mauricio Beccera, Liberty Teodoro, Bryan M. Spann, James Brewer, Helen Vanderswag, Adam Fleisher, Judith L. Heidebrink, Joanne L. Lord, Sara S. Mason, Colleen S. Albers, David Knopman, Kris Johnson, Rachelle S. Doody, Javier Villanueva-Meyer, Munir Chowdhury, Susan Rountree, Mimi Dang, Yaakov Stern, Lawrence S. Honig, Karen L. Bell, Beau Ances, John C. Morris, Maria Carroll, Mary L. Creech, Erin Franklin, Mark A. Mintun, Stacy Schneider, Angela Oliver, Daniel Marson, Randall Griffith, David Clark, David Geldmacher, John Brockington, Erik Roberson, Marissa Natelson Love, Hillel Grossman, Effie Mitsis, Raj C. Shah, Leyla deToledo-Morrell, Ranjan Duara, Daniel Varon, Maria T. Greig, Peggy Roberts, Marilyn Albert, Chiadi Onyike, Daniel D’Agostino, Stephanie Kielb, James E. Galvin, Brittany Cerbone, Christina A. Michel, Dana M. Pogorelec, Henry Rusinek, Mony J. de Leon, Lidia Glodzik, Susan De Santi, P. Murali Doraiswamy, Jeffrey R. Petrella, Salvador Borges-Neto, Terence Z. Wong, Edward Coleman, Charles D. Smith, Greg Jicha, Peter Hardy, Partha Sinha, Elizabeth Oates, Gary Conrad, Anton P. Porsteinsson, Bonnie S. Goldstein, Kim Martin, Kelly M. Makino, M. Saleem Ismail, Connie Brand, Ruth A. Mulnard, Gaby Thai, Catherine Mc-Adams-Ortiz, Kyle Womack, Dana Mathews, Mary Quiceno, Allan I. Levey, James J. Lah, Janet S. Cellar, Jeffrey M. Burns, Russell H. Swerdlow, William M. Brooks, Liana Apostolova, Kathleen Tingus, Ellen Woo, Daniel H.S. Silverman, Po H. Lu, George Bartzokis, Neill R. Graff-Radford, Francine Parfitt, Tracy Kendall, Heather Johnson, Martin R. Farlow, Ann Marie Hake, Brandy R. Matthews, Jared R. Brosch, Scott Herring, Cynthia Hunt, Christopher H. van Dyck, Richard E. Carson, Martha G. MacAvoy, Pradeep Varma, Howard Chertkow, Howard Bergman, Chris Hosein, Sandra Black, Bojana Stefanovic, Curtis Caldwell, Ging-Yuek Robin Hsiung, Howard Feldman, Benita Mudge, Michele Assaly, Elizabeth Finger, Stephen Pasternack, Irina Rachisky, Dick Trost, Andrew Kertesz, Charles Bernick, Donna Munic, Marek-Marsel Mesulam, Kristine Lipowski, Sandra Weintraub, Borna Bonakdarpour, Diana Kerwin, Chuang-Kuo Wu, Nancy Johnson, Carl Sadowsky, Teresa Villena, Raymond Scott Turner, Kathleen Johnson, Brigid Reynolds, Reisa A. Sperling, Keith A. Johnson, Gad Marshall, Jerome Yesavage, Joy L. Taylor, Barton Lane, Allyson Rosen, Jared Tinklenberg, Marwan N. Sabbagh, Christine M. Belden, Sandra A. Jacobson, Sherye A. Sirrel, Neil Kowall, Ronald Killiany, Andrew E. Budson, Alexander Norbash, Patricia Lynn Johnson, Thomas O. Obisesan, Saba Wolday, Joanne Allard, Alan Lerner, Paula Ogrocki, Curtis Tatsuoka, Parianne Fatica, Evan Fletcher, Pauline Maillard, John Olichney, Charles DeCarli, Owen Carmichael, Smita Kittur, Michael Borrie, T-Y Lee, Rob Bartha, Sterling Johnson, Sanjay Asthana, Cynthia M. Carlsson, Steven G. Potkin, Adrian Preda, Dana Nguyen, Pierre Tariot, Anna Burke, Nadira Trncic, Adam Fleisher, Stephanie Reeder, Vernice Bates, Horacio Capote, Michelle Rainka, Douglas W Scharre, Maria Kataki, Anahita Adeli, Earl A. Zimmerman, Dzintra Celmins, Alice D. Brown, Godfrey D. Pearlson, Karen Blank, Karen Anderson, Laura A. Flashman, Marc Seltzer, Mary L. Hynes, Robert B. Santulli, Kaycee M. Sink, Leslie Gordineer, Jeff D. Williamson, Pradeep Garg, Franklin Watkins, Brian R. Ott, Henry Querfurth, Geoffrey Tremont, Stephen Salloway, Paul Malloy, Stephen Correia, Howard J. Rosen, Bruce L. Miller, David Perry, Jacobo Mintzer, Kenneth Spicer, David Bachman, Nunzio Pomara, Raymundo Hernando, Antero Sarrael, Norman Relkin, Gloria Chaing, Michael Lin, Lisa Ravdin, Amanda Smith, Balebail Ashok Raj, Kristin Fargher

**Affiliations:** 1 Department of Neurology, Qingdao Municipal Hospital, School of Medicine, Qingdao University, Qingdao, China; 2 Department of Neurology, Qingdao Municipal Hospital, Nanjing Medical University, Qingdao, China; 3 College of Medicine and Pharmaceutics, Ocean University of China, China; 4 Department of Radiology, Qingdao Municipal Hospital, School of Medicine, Qingdao University, Qingdao, China; 5 Department of Neurology, Nanjing First Hospital, Nanjing Medical University, Nanjing, China; 6 Magnetic Resonance Unit at the VA Medical Center and Radiology, Medicine, Psychiatry and Neurology, University of California, San Francisco, USA; 7 San Diego School of Medicine, University of California, California, USA; 8 Mayo Clinic, Minnesota, USA; 9 Mayo Clinic, Rochester, USA; 10 University of California, Berkeley, USA; 11 University of Pennsylvania, Pennsylvania, USA; 12 University of Southern California, California, USA; 13 University of California, Davis, California, USA; 14 MPH Brigham and Women’s Hospital/Harvard Medical School; Massachusetts, USA; 15 Indiana University, Indiana, USA; 16 Washington University St. Louis, Missouri, USA; 17 Oregon Health and Science University, Oregon, USA; 18 University of California--San Diego, California, USA; 19 University of Michigan, Michigan, USA; 20 Baylor College of Medicine, Houston, State of Texas, USA; 21 Columbia University Medical Center, South Carolina, USA; 22 University of Alabama – Birmingham, Alabama, USA; 23 Mount Sinai School of Medicine, New York, USA; 24 Rush University Medical Center, Rush University, Illinois, USA; 25 Wien Center, Florida, USA; 26 Johns Hopkins University, Maryland, USA; 27 New York University, NY, USA; 28 Duke University Medical Center, North Carolina, USA; 29 University of Kentucky, Kentucky, USA; 30 University of Rochester Medical Center, NY, USA; 31 University of California, Irvine, California, USA; 32 University of Texas Southwestern Medical School, Texas, USA; 33 Emory University, Georgia, USA; 34 University of Kansas, Medical Center, Kansas, USA; 35 University of California, Los Angeles, California, USA; 36 Mayo Clinic, Jacksonville, USA; 37 Yale University School of Medicine, Connecticut, USA; 38 McGill University, Montreal-Jewish General Hospital, Canada.; 39 Sunnybrook Health Sciences, Ontario, USA; 40 U.B.C. Clinic for AD & Related Disorders, Canada.; 41 Cognitive Neurology - St. Joseph’s, Ontario, USA; 42 Cleveland Clinic Lou Ruvo Center for Brain Health, Ohio, USA; 43 Northwestern University, USA; 44 Premiere Research Inst (Palm Beach Neurology), USA; 45 Georgetown University Medical Center, Washington D.C, USA; 46 Brigham and Women’s Hospital, Massachusetts, USA; 47 Stanford University, California, USA; 48 Banner Sun Health Research Institute, USA; 49 Boston University, Massachusetts, USA; 50 Howard University, Washington D.C, USA; 51 Case Western Reserve University, Ohio, USA; 52 University of California, Davis – Sacramento, California, USA; 53 Neurological Care of CNY, USA; 54 Parkwood Hospital, Pennsylvania, USA; 55 University of Wisconsin, Wisconsin, USA; 56 University of California, Irvine – BIC, USA; 57 Banner Alzheimer’s Institute, USA; 58 Dent Neurologic Institute, NY, USA; 59 Ohio State University, Ohio, USA; 60 Albany Medical College, NY, USA; 61 Hartford Hospital, Olin Neuropsychiatry Research Center, Connecticut, USA; 62 Dartmouth-Hitchcock Medical Center, New Hampshire, USA; 63 Wake Forest University Health Sciences, North Carolina, USA; 64 Rhode Island Hospital, state of Rhode Island, USA; 65 Butler Hospital, Providence, Rhode Island, USA; 66 University of California, San Francisco, USA; 67 Medical University South Carolina, USA; 68 Nathan Kline Institute, Orangeburg, New York, USA; 69 Cornell University, Ithaca, New York, USA; 70 USF Health Byrd Alzheimer’s Institute, University of South Florida, USA

## Abstract

Phosphatidylinositolbinding clathrin assembly protein (PICALM) gene is one novel genetic player associated with late-onset Alzheimer’s disease (LOAD), based on recent genome wide association studies (GWAS). However, how it affects AD occurrence is still unknown. Brain reserve hypothesis highlights the tolerant capacities of brain as a passive means to fight against neurodegenerations. Here, we took the baseline volume and/or thickness of LOAD-associated brain regions as proxies of brain reserve capacities and investigated whether *PICALM* genetic variations can influence the baseline reserve capacities and the longitudinal atrophy rate of these specific regions using data from Alzheimer’s Disease Neuroimaging Initiative (ADNI) dataset. In mixed population, we found that brain region significantly affected by *PICALM* genetic variations was majorly restricted to posterior cingulate. In sub-population analysis, we found that one *PICALM* variation (C allele of rs642949) was associated with larger baseline thickness of posterior cingulate in health. We found seven variations in health and two variations (rs543293 and rs592297) in individuals with mild cognitive impairment were associated with slower atrophy rate of posterior cingulate. Our study provided preliminary evidences supporting that *PICALM* variations render protections by facilitating reserve capacities of posterior cingulate in non-demented elderly.

The global situation of dementia is not optimistic. The prevalence of dementia was estimated 5–7% in most global regions and 35.6 million people lived with dementia in 2010, with numbers predicted to almost double every 20 years, to 65.7 million in 2030 and 115.4 million in 2050^1^,^2^, leading to an increasing burden on caregivers and society^3^. The recently released Alzheimer Report 2015 reflects a same trend but lousier prospect. (http://www.alzforum.org/news/research-news/world-alzheimer-report-2015-revised-estimates-hint-larger-epidemic) As the most common type (roughly 60%) of dementia, Alzheimer’s disease (AD) significantly inflicts both reduced life-span and lowered life quality on patients^4^,^5^,^6^.

In confrontation of this situation, scientific efforts to elucidate its etiology has never been stopped. It is now widely accepted that AD is a complex disease entity, with occurrence underpinned by both genetic and environmental components^7^,^8^. *APOE4* was a widely validated genetic risk but merely accounted for a limited percentage of LOAD risk, several genome-wide association studies (GWAS) and meta-analyses had revealed a series of new risk loci associated with the late-onset type of AD (LOAD; >65 years of age)^9^,^10^,^11^,^12^, to some extent filling up the vacant area of its genetic etiology.

The gene encoding phosphatidylinositolbinding clathrin assembly protein (PICALM) was one of these new players. Its association with AD was revealed in large GWAS^9^,^10^,^11^,^12^ and further validated in a series of larger replication studies in both European^13^,^14^,^15^,^16^,^17^ and Asian population^18^, in spite of some conflicting results from those with smaller sample sizes^19^. However, the concrete pathways by which PICALM gene are involved in AD occurrence are still an enigma.

More than a decade ago, Stern^20^,^21^ proposed the concept of reserve to explain the disjunction between AD pathology degree and severity of clinical performances. The hypothesis proposed a passive protective model named “brain reserve”, positing that the quantity of available neural substrate (e.g., brain size, synaptic count, or dendritic branching) can be the basis of cerebral tolerance to abnormal insults (Fig. 1A)^8^,^22^. However, it seemed that previous understandings put more focus on the global situation of the whole brain than on some brain regions specifically associated with the disease, such as hippocampus (CA1 subregion), middle temporal area, entorhinal area, posterior cingulate, precuneus, and parahippocampal area. Based on these findings, we thus supposed that LOAD-associated genetic variations may be involved in AD occurrence by modulating the brain reserve capacities of these brain sub-regions which has been proved vulnerable in AD process (Fig. 1B).

Herein, we took the baseline volume and/or thickness of AD-associated brain regions (as mentioned above) as proxies of brain reserve capacities and investigated whether *PICALM* genetic variations can influence the reserve capacities and longitudinal atrophy rate of these specific regions using data from Alzheimer’s Disease Neuroimaging Initiative (ADNI) dataset.

## Results

### Demographic, cognitive, and clinical characteristics

Demographic, cognitive, and clinical characteristics of the included subjects are shown in Table 1. In brief, a total of 281 NC (145 female, 74.51 ± 5.56 years), 483 MCI (201 female, 72.28 ± 7.45 years) and 48 AD patients (18 female, 75.51 ± 9.23 years) were enrolled in the present study. The frequency for the ε4 allele of APOE gene was AD > MCI > NC. For the cognitive function, AD patients displayed the worst cognitive function according to various neuropsychological scales, including CDRSB, MMSE, ADAS-cog, RAVLT, FAQ and MoCA. For the brain reserve capacity, AD patients showed the most severe atrophy in hippocampus, middle temporal and entorhinal cortex.

### Brain structures and *PICALM* genotypes in the mixed population

At baseline, no loci showed significant association with volume of either hippocampus or hippocampal CA1 region. A allele of rs3851179 showed trend of association with larger thickness of right entorhinal area. G allele of rs561655 showed trend of association with larger thickness of parahippocampal region. C allele of rs592297 showed trend of association with smaller volume of left middle temporal area and larger thickness of parahippocampal region. C allele of rs642949 showed trends of association with larger volume of left middle temporal area and right posterior cingulate, larger thickness of left precuneus and smaller thickness of left parahippocampal area. However, all these associations failed to survive the FDR correction (Fig. 2 and Supplementary Table 2).

Analysis after one year follow-up indicated faster atrophy rate of right hippocampal CA1 for individuals carrying variations in rs543293 (A allele) and rs1237999 (G allele). C allele of rs64249 showed trends of associations with slower atrophy rate of left hippocampus and faster atrophy rate of right precuneus. Nonetheless, these associations did not reach significant after FDR correction. Interestingly, we found slower atrophy rate of right posterior cingulate in individuals with variations of rs561655 (G allele), rs543293 (A allele), rs592297 (C allele), rs1237999 (G allele) and 7941541 (G allele) and faster atrophy rate of the same region in individuals with variation of rs642949 (C allele) The associations were still significant after FDR correction (Fig. 3A–F).

After two years follow-up, no loci showed significant association with atrophy rate of hippocampus or hippocampal CA1 region. Atrophy rate of middle temporal area showed trend of association with variation of rs642949. Atrophy rate of posterior cingulate showed trend of association with variation of rs3851179, rs543293, rs7941541, and rs642949. Atrophy rate of precuneus showed trend of association with variation of rs561655 and rs642949. Atrophy rate of parahippocampal area showed trend of association with variation of rs642949. Nonetheless, none of these reached significance after FDR correction, possibly due to the shrunken sample size after two years follow-up (Fig. 2 and Supplementary Table 2).

Altogether, we can infer that posterior cingulate may be the pivotal region on which *PICALM* variations target. Further, we selected the posterior cingulate as our sole ROI and independently tested its association with *PICALM* variations in NC and MCI individuals, respectively.

### Posterior cingulate and *PICALM* genotypes in NC individuals

The associations of variations in four *PICALM* loci (rs561655, rs1237999, rs543293 and rs592297) with slower one-year atrophy rate of posterior cingulate were further validated in the NC population (Fig. 4B–F). Interestingly, we found that one *PICALM* variation (C allele of rs642949) was associated with larger thickness of posterior cingulate at baseline (Fig. 4A). We found significant association of rs561655, rs7941541 and rs3851179 with slower two years atrophy rate of posterior cingulate (Fig. 4G–I). This is expectable since the major contributors to brain atrophy and atrophy rate differ between NC individuals and MCI/AD individuals, such that the overall atrophy rate of posterior cingulate in the mixed population (NC+MCI+AD) was faster than that in the NC population (Fig. 4L).

### Posterior cingulate and *PICALM* genotypes in MCI individuals

Compared to NC population, the trend of associations of *PICALM* variations with atrophy rate of posterior cingulate in MCI population were consistent (Fig. 2). We found A allele of rs543293 and C allele of rs592297 were associated with slower atrophy rate (after one year and two years) of posterior cingulate, respectively (Fig. 4J,K).

### Posterior cingulate and *PICALM* genotypes in AD individuals

We failed to identify any significant associations of reserve capacities of posterior cingulate with *PICALM* variations in AD population, possibly due to the constrained sample size.

## Discussion

**Figure 1:**
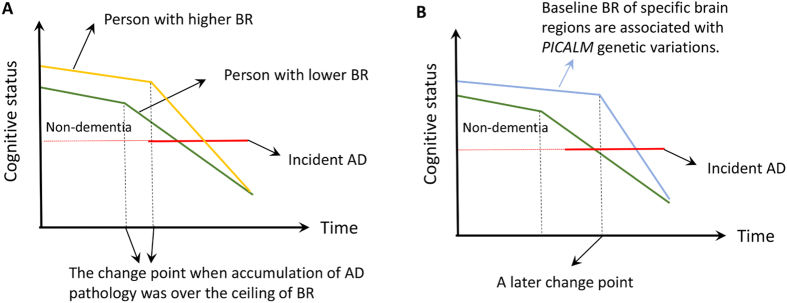
(**A**) Depiction of how brain reserve operates to protect the brain. The x-axis represents time, over which AD pathology slowly accumulates. The y-axis represents cognitive function. We assume that AD pathology accumulates over time at the same rate in two individuals with high and low brain reserve (BR). The amount of pathology needed before cognitive function is affected is greater for individual with higher CR, leading to a later change point of time. It follows that greater pathology will be needed for the person with higher BR to meet clinical diagnostic criteria for AD, thus delaying the onset of the disease. Once cognitive decline arises, it is faster in the person with higher BR^22^. (**B**) We proposed a hypothesis that *PICALM* genetic variations were associated with brain reserve (baseline thickness/volume and atrophy rate) of specific regions associated with AD in non-dementia elderly. We hypothesized that individuals carrying specific *PICALM* variations might have higher baseline thickness/volume of specific brain areas and/or slower atrophy rate in confrontation with impacts of pathological impairments and/or normal aging for some unknown reasons. Based on model depicted in (**A**), these trends equivalently render two powerful “weapons” for the individuals to maintain normal cognition and stay away from AD over a period longer than others. Abbreviation: BR = brain reserve; AD = Alzheimer’s disease.

**Figure 2:**
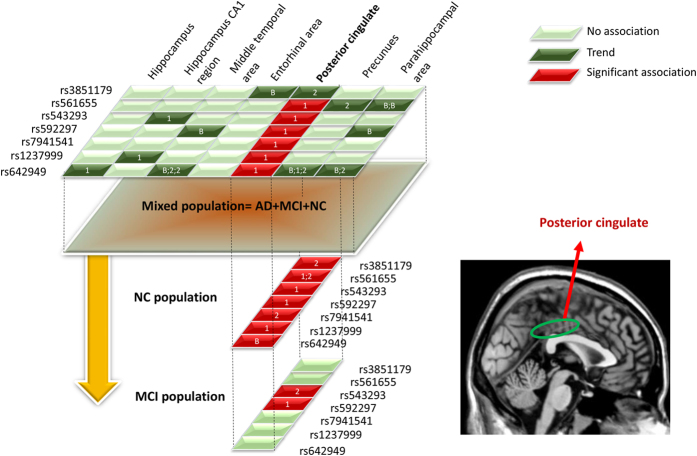
The procedure and gross results of associations analysis in mixed population and sub-population (NC and MCI). In mixed population, we identified multiple brain regions showing trend of association with PICALM genetic variations (Deep green block). However, only posterior cingulate survived the FDR correction (Red block); We further tested this genetic predisposition in NC and MCI population. Results in NC individuals indicated that variations of seven loci were associated with baseline or one year or two years atrophy rate of posterior cingulate; Results in MCI individuals indicated that variations of two loci were associated with one year or two years atrophy rate of posterior cingulate. Abbreviations: NC = normal cognition; MCI = mild cognition impairment; AD = Alzheimer’s disease.

**Figure 3:**
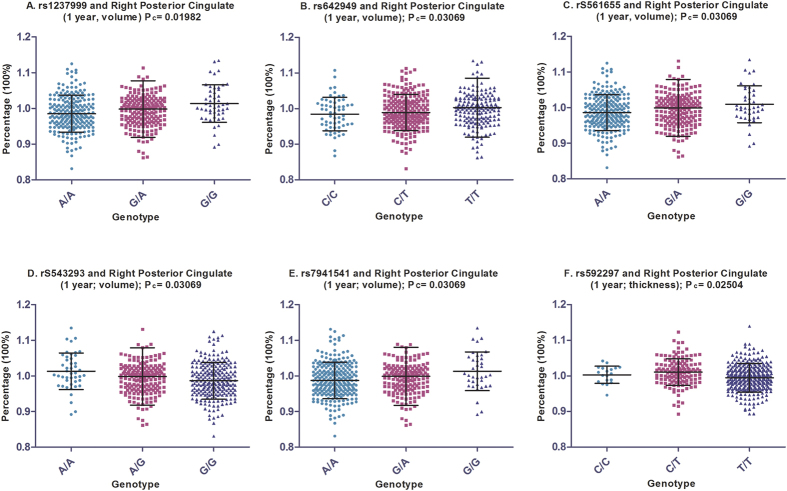
The significant associations of *PICALM* loci with atrophy rate of posterior cingulate in the mixed population. We identified six loci which associations were still significant after FDR correction in the mixed population. The (**A–F**) depicted that variations of rs1237999, rs642949, rs561655, rs543293, rs7941541 and rs592297 were associated with one-year atrophy rate of posterior cingulate.

We present here an explorative study about how single nucleotide polymorphisms of *PICALM* impart influences on brain reserve capacity of AD-associated brain regions. Seven SNPs were finally included in the analysis. We found six loci (all except rs3851179) in mixed population, two loci (rs592297 and rs543293) in MCI population and all seven loci in NC population, which were significantly associated with higher baseline thickness and/or slower atrophy rate of posterior cingulate, both of which would be favorable in fighting against AD insults, despite in a passive manner (Fig. 1B) These findings were significant given that 1) our study further revealed the potential pathways by which these genetic variations act in protecting brain from AD; 2) Our findings confirmed the protective roles of certain loci of PICALM gene, which is consistent with previous meta-analysis results of association of AD with rs561655 (odd ratio [OR] = 0.87; 95% confidence interval [95% CI] = 0.83–0.92) and rs543293 (OR = 0.89; 95% CI = 0.85–0.94). (http://www.alzgene.org/meta.asp?geneID=636)

**Figure 4:**
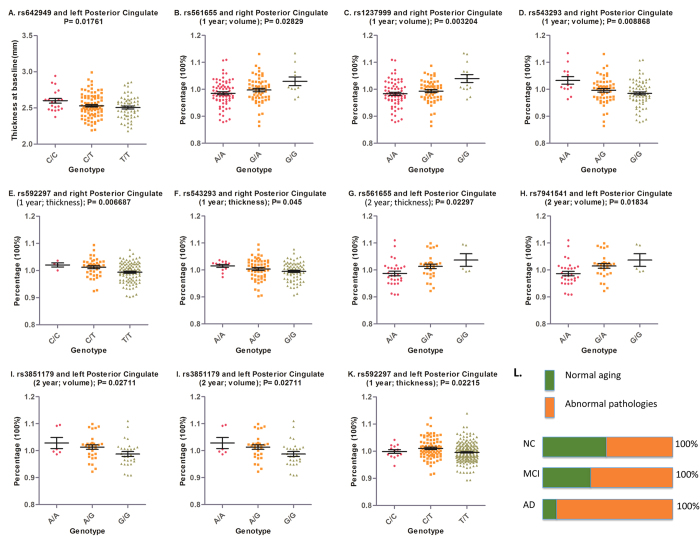
The significant associations of *PICALM* loci with baseline thickness and atrophy rate of posterior cingulate in health and MCI individuals. (**A**) Depicted that rs642949 (C allele) was associated with larger thickness of posterior cingulate in NC population; (**B–I**) Depicted that variations of rs561655, rs1237999, rs543293, rs592297, rs7941541 were associated with slower atrophy rate of posterior cingulate in NC population; (**J,K**) Depicted that rs543293 and rs592297 were associated with slower atrophy rate of posterior cingulate in MCI population. (**L**) Depicted that the contributors to brain atrophy majorly included normal aging and pathological insults. The proportion of the latter would arise constantly as the stage progresses (from NC to MCI to AD) and finally become the predominant factor. This may explain the difference of association which *PICALM* genetic variations showed in NC and MCI population. Abbreviations: NC = normal cognition; MCI = mild cognition impairment; AD = Alzheimer’s disease.

Though our study showed that *PICALM* variations were associated with higher brain reserve capacities of posterior cingulate, the mechanism was still a mystery. Generally, the major contributors to brain atrophy include normal aging in which the process is relatively slower and stable, as well as abnormal pathological insults in which the process is relatively faster and changeable. In NC individuals, cerebral resistance power is enough to tolerate these two adverse contributors, thus contributing to the normal cognitive functions. Although we know little about which contributor held a dominant position in inducing brain atrophy in the NC stage, it is reasonably inferred that the role of abnormal pathologies is increasingly rising and would finally surpass that of normal aging as the stage further progresses (for example, from NC to MCI/AD) (Fig. 4L). In the present analysis, we found that *PICALM* genetic variations were more inclined to be associated with atrophy rate in NC individuals. Given that normal aging might play a more important role in causing brain atrophy in the stage of NC than MCI/AD, it can be thus inferred that the potential pathways by which *PICALM* variations act may be possibly associated with fighting against normal aging of posterior cingulate. More researches warrant to validate this hypothesis.

On the other hand, posterior cingulate cortex is located in the medial part of the inferior parietal lobe and lies within the posteromedial cortex. This specific brain area is highly anatomically connected and is known as a pivotal part of the default mode network (DMN), which is a resting-state functional networks and is particularly active in healthy people when they do not think about anything (for review see^23^,^24^). Previous cross-sectional analysis suggested that both AD and MCI subjects showed significant difference of posterior cingulate when compared with the health^25^,^26^,^27^. Also, disorder of DMN was a characteristic feature seen in early AD^24^. All these findings were suggestive of an impellent role of neurodegeneration of posterior cingulate in the very early stage of AD^28^. Our study provided the first evidence linking *PICALM* genetic variations with slower atrophy rate of posterior cingulate, leading to a reasonable postulation that individuals carrying these specific variations would be less vulnerable to lower reserve capacity of posterior cingluate and be possibly thus more powerful in fighting neurodegenerative insults.

Furthermore, as a critical network of brain, DMN was composed of large amounts of communication hubs named “synapses”. A very recent study proposed that DMN correlated with the orchestrated activity of dozens of genes linked to ion channel activity and synaptic function^29^, emphasizing the importance of synapses in maintaining normal functions of this network. On the other hand, it was previously reported that abnormalities of synapses in posterior cingulate occurred in the early stage of AD^30^. Therefore, PICALM may protect the normal operations of synapse by facilitating neurotransmitter delivering, which is against the negative impacts derived from normal aging or pathological insults such as Aβ. (for review see^31^)

Several limitations exist in our study. First of all, the sample size in the present analysis was smaller than that in the traditional large GWAS studies (n > 10,000); Second, the follow-up was relatively short. Both of these lead to restricted power and thus restrain our making definite conclusions. Therefore, this study is only a preliminary investigation and future replication with larger sample size and longer follow-up is necessary. Third, not all SNPs of PICALM gene were included due to the restriction of ADNI database. Fourth, associations of PICALM gene with posterior cingulate in AD population need more work given the AD sample in our study is obviously constrained. These may lead to insufficient digging of influences of *PICALM* genetic variations and future research warrant. Fifth, it is noteworthy that the results from sub-population (NC or MCI or AD) may be more informative than those from mixed population, which lead to, again, necessities of future efforts with larger sample.

In summary, this study provided preliminary evidences supporting that *PICALM* variations render protections by facilitating reserve capacities of posterior cingulate in non-demented elderly.

## Materials and Methods

### Definition of brain reserve (BR)

Brain reserve (BR) can be metaphorized as cerebral pre-existing troops (such as brain/specific brain region size, neuron/synaptic count, and dendritic branching, etc.), which are wholeheartedly responsible for maintaining a normal cognitive function by passively defending against attacks from pathological insults (for example, Alzheimer’s disease) as well as normal aging. However, once the loss of these troops achieved a certain level (so-called threshold model), cognitive impairments occurred.

### ADNI database

Alzheimer’s Disease Neuroimaging Initiative (ADNI) is a large, multicenter, longitudinal neuroimaging study, initiated in 2003 by the National Institute on Aging, the National Institute of Biomedical Imaging and Bioengineering, the Food and Drug Administration, private pharmaceutical companies, and nonprofit organizations^32^. The initial goal of ADNI was to recruit 800 subjects but the ADNI has been further followed by ADNI-GO and ADNI-2. To date, the three protocols have recruited over 1,500 adults (aged 55 to 90), consisting of cognitively normal older individuals, people with early or late MCI, and people with AD. The study was approved by the institutional review boards of all participating centers, and written informed consent was obtained from all participants or authorized representatives after extensive description of the ADNI according to the 1975 Declaration of Helsinki^33^. The study was approved by the institutional review boards of all participating centers (Ocean University of China, Qingdao Municipal Hospital, Nanjing First Hospital, Memory and Aging Center in University of California, and ADNI) and written informed consent was obtained from all participants or authorized representatives. In addition, the methods were carried out in accordance with the approved guidelines.

### Participants

The data used in this study were obtained from the ADNI database (http://adni.loni.usc.edu) Inclusion criteria for AD subjects is National Institute of Neurological and Communication Disorders/Alzheimer’s Disease and Related Disorders Association (NINCDS/ADRDA) criteria for probable AD, with a Mini Mental State Examination (MMSE) score between 20 and 26, a global Clinical Dementia Rating (CDR) of 0.5 or 1, a sum-of-boxes CDR of 1.0 to 9.0. All amnestic MCI subjects fulfilled a MMSE score of 24 to 30 and a Memory Box score of at least 0.5. Otherwise, the subjects who had any serious neurological disease other than possible AD, or any history of brain lesions or head trauma, or were psychoactive medication user (including antidepressants, neuroleptics, chronic anxiolytics, or sedative hypnotics) were excluded. More details concerning the ADNI cohort were reported elsewhere^32^,^34^. The final dataset for the present analysis comprised 812 individuals, including 281 health controls (normal cognition, NC), 483 MCI and 48 AD at baseline. The basic data of subjects in our analysis was downloaded from the ADNI website in 2015.

### Genetic data and SNP selection

Bead Studio 3.2 software and a recent Genome Studio v2009.1 (Illumina) were successively used to generate SNP genotypes from bead intensity data^35^. Additionally, the widely used PLINK data format was accessible to facilitate analysis by other groups. In our study, *PICALM* genotypes were extracted from the ADNI PLINK data format and the quality control procedures were performed using PLINK software. Filtering criteria applied to individuals and SNPs were as follows: minimum call rates >90%, minimum minor allele frequencies (MAF) >0.05, Hardy-Weinberg equilibrium test P > 0.001 (Table 2).

SNPs reported to be significantly associated with AD by GWASs^9^,^10^,^12^ were preferentially selected for analysis. As supplementary strategy, we further searched the potentially promising *PICALM* SNPs from meta-analysis and replication studies^15^,^36^,^37^,^38^,^39^. A total of 22 SNPs (Supplementary Table 1) were initially identified in the initial screening, among which 15 SNPs were further excluded, including 12 not found in ADNI and 3 with a MAF < 0.05 (Fig. S1). Finally, we chose the remaining 7 loci as our target SNPs in this study (Table 2).

### MRI structure

ADNI MRIs were acquired at multiple sites with a GE Healthcare (Buckinghamshire, England), Siemens Medical Solutions USA (Atlanta, Georgia), or Philips Electronics 3.0 T system (Philips Electronics North America; Sunnyvale, California)^40^. These analyses utilized the dataset of UCSF FreeSurfer to conduct association test of *PICALM* genotypes with brain structure. We processed the cerebral image segmentation and analysis using the FreeSurfer version 5.1.0 software package (http://surfer.nmr.mgh.harvard.edu/) based on the 2010 Desikan-Killany atlas^41^. The main work contained that motion correction and averaging of multiple volumetric T1-weighted images (when more than one is available)^42^, removal of non-brain tissue using a hybrid watershed/deformable surface algorithm^43^, automated Talairach transformation, segmentation of the subcortical white matter and deep gray matter volumetric structures (including hippocampus, amygdala, caudate, putamen, ventricles)^44^,^45^, intensity normalization^46^, tessellation of the gray matter white matter boundary, automated topology correction^47^, and surface deformation following intensity gradients to optimally place the gray/white and gray/cerebrospinal fluid (CSF) borders at the location where the greatest shift in intensity defines the transition to the other tissue class^48^. More detailed technical procedures were available in previous study^48^.

Here, we defined seven brain regions, including hippocampus, hippocampus CA1 subregion, middle temporal area, entorhinal area, posterior cingulate, precuneus and parahippocampal area, as regions of interest (ROIs). These regions were known to be affected by AD and their atrophy in AD has been previously validated via MRI studies^49^,^50^,^51^,^52^,^53^. In the present analysis, there were 812 (NC = 281, MCI = 483, AD = 48) individuals included in the regional volume/thickness analysis (Table 1).

### Statistical analysis

Differences in continuous variables were examined using one-way analysis of variance (ANOVA), and categorical data were tested using chi-square test. Furthermore, a multiple linear regression model which considered age, gender, education, intracranial volume and ApoE4 status as covariates was used to estimate the possible correlation between volume/thickness (baseline data and follow-up changes) and *PICALM* genotypes. All statistical analyses were performed by R 3.12 (http://www.r-project.org/) and PLINK 1.07 (http://pngu.mgh.harvard.edu/wpurcell/plink/). As Bonferroni correction was inappropriate owing to the nonindependence of tests^40^, we used the false discovery rate (FDR), the method developed by Hochberg and Benjamini^54^, to control for multiple hypothesis testing. The criterion for significant difference was P < 0.05 according to FDR correction.

We first screened significant brain regions associated with *PICALM* loci in the mixed population comprising individuals with normal cognition (NC), mild cognitive impairment (MCI) and Alzheimer’s disease. To further validate the hereditary susceptibility in different population, we then repeated the test independently using sub-population, including NC and MCI and AD individuals.

## Additional Information

**How to cite this article**: Xu, W. *et al.* The impact of *PICALM* genetic variations on reserve capacity of posterior cingulate in AD continuum. *Sci. Rep.* **6**, 24480; doi: 10.1038/srep24480 (2016).

## Supplementary Material

Supplementary Information

## Figures and Tables

**Table 1:** The characteristics of the ADNI subjects at baseline.

Characteristics	NC		MCI		AD		P^*^
Age (years)	281	74.51 ± 5.56	483	72.28 ± 7.45	48	75.51 ± 9.23	<0.01
Gender (male/female)	281	136/145	483	282/201	48	30/18	0.02
Education (years)	281	16.41 ± 2.66	483	15.98 ± 2.82	48	15.73 ± 2.62	0.08
APOE ε4 (0/1/2)	281	204/70/7	483	262/180/41	48	14/25/9	<0.01
CDR-SB	207	0.03 ± 0.13	406	1.44 ± 0.87	47	4.44 ± 1.69	<0.01
MMSE	281	29.07 ± 1.15	483	27.89 ± 1.69	48	22.96 ± 2.03	<0.01
ADAS-cog	281	9.06 ± 4.23	480	15.30 ± 6.65	48	29.80 ± 8.44	<0.01
RAVLT	280	44.83 ± 9.60	483	36.16 ± 10.86	47	22.32 ± 7.84	<0.01
FAQ	281	0.17 ± 0.66	481	2.85 ± 3.99	48	12.6 ± 7.14	<0.01
Hippocampus (mm^3^)	257	7344 ± 895	422	6996 ± 1126	39	5757 ± 948	<0.01
Middle Temporal (mm^3^)	257	20298 ± 2600	422	20186 ± 2735	39	17776 ± 3230	<0.01
Entorhinal (mm^3^)	257	3803 ± 650	422	3610 ± 723	39	2919 ± 705	<0.01
CMRgl	207	6.55 ± 0.55	406	6.32 ± 0.64	47	5.30 ± 0.72	<0.01
SUVR	152	1.12 ± 0.19	323	1.20 ± 0.22	46	1.39 ± 0.22	<0.01

NC, normal cognition; MCI, mild cognition impairment; AD, Alzheimer’s disease; CDR-SB, Clinical Dementia Rating sum of boxes; ADAS-cog, Alzheimer’s disease Assessment Scale Cognition; MMSE, Mini-Mental State Exam; RAVLT, Rey Auditory Verbal Learning Test; FAQ, Functional Activities Questionnaire; CMRgl, Cerebral Metabolism Rate for glucose measured with fluorodeoxyglucose-positron emission tomography (FDG-PET). SUVR, florbetapir standard uptake value ratios on amyloid imaging.

^*^P values for continuous variables are from one-way analysis of variance (ANOVA). P values for categorical data are from chi square test.

Data are given as mean ± standard deviation unless otherwise indicated.

**Table 2:** Characteristics of seven SNPs finally selected for our analysis.

N	SNP	Chr	Allele change	Position	SNP source	H-W p value	Reference	MAF#
1	rs3851179	11	G→A	5′ downstream	GWAS	0.9485	[Harold^9^] [Seshadri^10^]	0.3149
2	rs561655	11	A→G	5′ downstream	GWAS & Meta-analysis	0.8524	[Lambert^11^] [Jun^15^]	0.3407
3	rs543293	11	G→A	5′ downstream	Replication	0.625	[Lee^15^] [Jun^36^]	0.2923
4	rs592297	11	T→C	Exon 5	This SNP is associated with specific PICALM isoform expression level and in strong LD with rs3851179 and is a part of an exonic splice enhancer region in exon 5	0.8578	[Parikh^37^] [Schnetz^38^]	0.2113
5	rs7941541	11	A→G	5′ downstream	Replication study	0.7482	[Lee^36^]	0.289
6	rs1237999	11	A→G	5′ downstream	GWAS	0.8185	[Harold^9^]	0.3297
7	rs642949	11	T→C	Intron region of NM_001008660.2	This SNP is in strong LD with rs592997	0.4931	[Furney^39^]	0.4459

Abbreviation: MAF = Minor Allele Frequency; SNP = Single Nucleotide Polymorphism; H-W = Hardy-Weinberg.

^#^MAF data was calculated by Haploview 4.2.
