# Supplementary material for: The impact of PICALM genetic variations on reserve capacity of posterior cingulate in AD continuum
Source: Sci Rep. 2016 Apr 27;6:24480. doi: 10.1038/srep24480 (PMC4846810; doi:10.1038/srep24480)
Supplement: Supplementary Information [file srep24480-s1.doc]

**The impact of PICALM genetic variations on reserve capacity of posterior cingulate in AD continuum**

Wei Xu^1,a^, Hui-Fu Wang^2,a^, Lin Tan^3^, Meng-Shan Tan^1^, Chen-Chen Tan^1^, Xi-Chen Zhu^2^, Dan Miao ^1^, Wan-Jiang Yu^4^, Teng Jiang^5^, Lan Tan^1,2,3^,*, Jin-Tai Yu^1,6^,*, Alzheimer’s Disease Neuroimaging Initiative

^1^ Department of Neurology, Qingdao Municipal Hospital, School of Medicine, Qingdao University, Qingdao, China

^2^ Department of Neurology, Qingdao Municipal Hospital, Nanjing Medical University, Qingdao, China

^3^ College of Medicine and Pharmaceutics, Ocean University of China, China

^4^ Department of Radiology, Qingdao Municipal Hospital, School of Medicine, Qingdao University, Qingdao, China

^5^ Department of Neurology, Nanjing First Hospital, Nanjing Medical University, Nanjing, China

^6^ Memory and Aging Center, Department of Neurology, University of California, San Francisco, CA, USA

a The first two authors contributed equally to this work.

* Address correspondence to Dr Lan Tan MD, PhD, Department of Neurology, Qingdao Municipal Hospital, School of Medicine, Qingdao University, No.5 Donghai Middle Road, Qingdao, Shandong Province 266071, China; or Jin-Tai Yu, MD, PhD, Department of Neurology, University of California, San Francisco, 675 Nelson Rising Lane, Suite 190, Box 1207, San Francisco, CA 94158, USA.

E-mail addresses: dr.tanlan@163.com (L. Tan); yu-jintai@163.com or jintai.yu@ucsf.edu (J.T. Yu);

Tel: +86-532-8890-5659; Fax: +86-532-8890-5659.

**Supplementary table 1- Information of SNP in the process of SNP sele**ction

| **N** | **SNP** | **Chr** | **Allele change** | **Position** | **SNP source** | **Reference (PMID)** | **Inclusion** | **MAF^#^** |
| --- | --- | --- | --- | --- | --- | --- | --- | --- |
| **1** | rs3851179 | 11 | G→A | 5' downstream | Two GWAS studies | [19734902]; [20460622] | **Yes** | 0.3149 |
| **2** | rs561655 | 11 | A→G | 5' downstream (close to rs543293) | GWAS; Meta-analysis | [21460841]; [20697030] | **Yes** | 0.3407 |
| **3** | rs543293 | 11 | G→A | 5' downstream (close to rs561655) | Two replication studies | [21059989]; [20697030] | **Yes** | 0.2923 |
| **4** | rs592297 | 11 | T→C | Exon 5 | This SNP is asociated with specific PICALM isoform expression level and in strong LD with rs3851179 and is a part of an exonic splice enhancer region in exon 5 | [24618820]; [22943764] | **Yes** | 0.2113 |
| **5** | rs7941541 | 11 | A→G | 5' downstream | Replication study | [21059989] | **Yes** | 0.289 |
| **6** | rs1237999 | 11 | A→G | 5' downstream | GWAS | [2845877] | **Yes** | 0.3297 |
| **7** | rs642949 | 11 | T→C | Intron region of NM_001008660.2. | This SNP is in strong LD with rs592997 | [21116278] | **Yes** | 0.4459 |
| 8 | rs17148827 | 11 | ∕ | ∕ | Replication | [22159054] | No | < 0.05 |
| 9 | rs12800974 | 11 | ∕ | ∕ | This SNP has potentially functional impact | [22960267] | No | 0 |
| 10 | rs17148741 | 11 | ∕ | ∕ | Meta-analysis | [22745009] | No | 0 |
| 11 | rs677909 | 11 | ∕ | ∕ | Replication | [22975751] | No | **NF** |
| 12 | rs536841 | 11 | ∕ | ∕ | GWAS | [2845877] | No | **NF** |
| 13 | rs17159904 | 11 | ∕ | ∕ | Replication | [21059989] | No | **NF** |
| 14 | rs596864 | 11 | ∕ | ∕ | Meta-analysis | [22745009] | No | **NF** |
| 15 | rs10792832 | 11 | ∕ | ∕ | GWAS-meta | [24162737] | No | **NF** |
| 16 | rs588076 | 11 | ∕ | ∕ | Associated with PICALM expression level | [25169757] | No | **NF** |
| 17 | rs17817992 | 11 | ∕ | ∕ | Meta-analysis | [22832961] | No | **NF** |
| 18 | rs12790526 | 11 | ∕ | ∕ |  |  | No | **NF** |
| 19 | rs12795381 | 11 | ∕ | ∕ |  |  | No | **NF** |
| 20 | rs12802399 | 11 | ∕ | ∕ |  |  | No | **NF** |
| 21 | rs541458 | 11 | ∕ | ∕ | Four replication studies | [21059989]; [20534741]; [21220176]; [20697030] | No | **NF** |
| 22 | rs12795381 | 11 | ∕ | ∕ | Replication | [22159054] | No | **NF** |
|  | ^#^ MAF data was calculated using Haploview software.  N1- N7 represents the SNPs finally selected out in this study.  **Abbreviation:** NF= Not Found in ADNI database; MAF= Minor Allele Frequency; SNP= Single Nucleotide Polymorphism; | | | | | | | |

**Figure S1. The SNPs included in the present analysis and its selection process.**

**
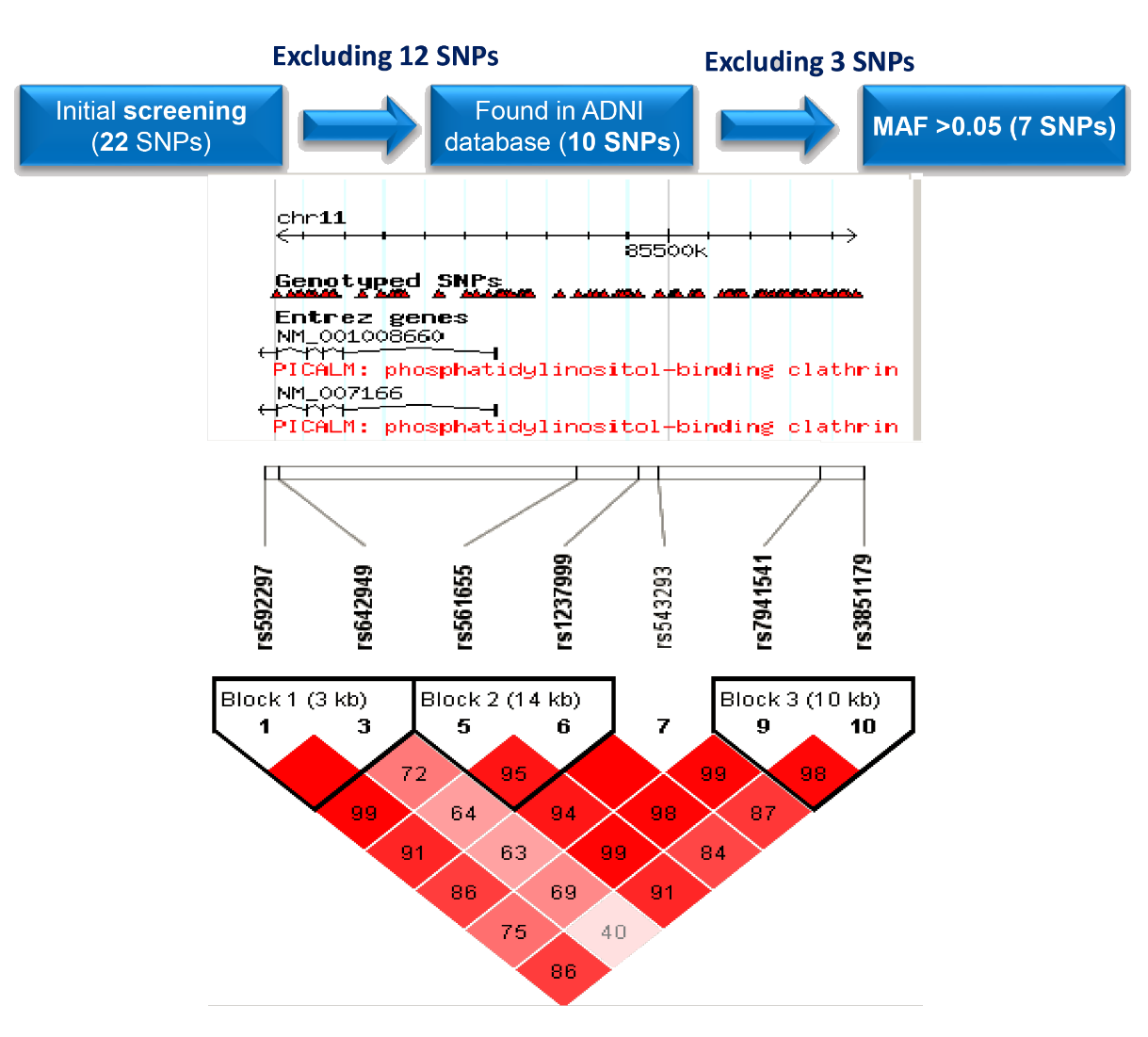
**

**Supplementary table 2A Gross results of relationship between *PICALM* variants and AD endophenotypes in hybrid population**

|  | | **Seven SNPs selected for analysis** | | | | | | |
| --- | --- | --- | --- | --- | --- | --- | --- | --- |
|  |  | **rs3851179** | **rs561655** | **rs543293** | **rs592297** | **rs7941541** | **rs1237999** | **rs642949** |
| ROIs associated with AD | Hippocampus | ∕ | ∕ | ∕ | ∕ | ∕ | ∕ | **☆** |
|  | Hippocampus (CA1) | ∕ | ∕ | **☆** | ∕ | ∕ | **☆** | ∕ |
|  | Middle TeOmporal | ∕ | ∕ | ∕ | ★ | ∕ | ∕ | ★●● |
|  | Entorhinal | ★ | ∕ | ∕ | ∕ | ∕ | ∕ | ∕ |
|  | **Posterior Cingulate** | ● | **☆** | **☆**● | **☆☆** | **☆**● | **☆** | ★**☆** |
|  | Precuneus | ∕ | ● | ∕ | ∕ | ∕ | ∕ | ★**☆**● |
|  | Parahippocampal | ∕ | ★★ | ∕ | ★ | ∕ | ∕ | ★● |

★ Baseline; **☆** 1 year of follow-up; ● 2 year of follow-up; Red color means SNP which is still significant after FDR correction;

**Abbreviation**: CA1= Cornu Ammonis 1; ROI= Region of Interest; SNP= Single Nucleotide Polymorphism

**Supplementary table 2B Detailed results of the associations of *PICALM* loci with brain regions on MRI in hybrid population**

| **ROI** | **SNP** | **Baseline** | | | **Follow-up (1 year)** | | | **Follow-up (2 year)** | | |
| --- | --- | --- | --- | --- | --- | --- | --- | --- | --- | --- |
|  |  | **Sample (L/R)** | **P-value (L/R)** | **Pc (L/R)** | **Sample (L/R)** | **P-value (L/R)** | **Pc (L/R)** | **Sample (L/R)** | **P-value (L/R)** | **Pc (L/R)** |
| Entorhinal (volume) | rs3851179 | 535/535 | 0.1148/0.09838 | 0.6711/0.6083 | 463/463 | 0.6385/0.7454 | 1/0.9317 | 238/238 | 0.4309/0.4668 | 0.8165/0.9536 |
|  | rs561655 | 536/536 | 0.1881/0.08948 | 0.6711/0.6083 | 464/464 | 0.9618/0.5681 | 1/0.8734 | 238/238 | 0.4638/0.7466 | 0.8165/1 |
|  | rs592297 | 536/536 | 0.7140/0.8178 | 0.8094/0.6871 | 464/464 | 0.8586/0.6114 | 1/0.8734 | 238/238 | 0.5609/0.1832 | 0.8165/0.9536 |
|  | rs7941541 | 536/536 | 0.3101/0.1663 | 0.6711/0.7666 | 464/464 | 0.8604/0.4512 | 1/0.8734 | 238/238 | 0.2709/0.9991 | 0.8165/0.9536 |
|  | rs1237999 | 535/535 | 0.3177/0.2028 | 0.6711/0.7954 | 463/463 | 0.9409/0.3493 | 1/0.8734 | 237/237 | 0.5715/0.5722 | 0.8165/0.9536 |
|  | rs543293 | 534/534 | 0.4027/0.3583 | 0.6711/0.9302 | 462/462 | 0.6362/0.4955 | 1/0.8734 | 236/236 | 0.4788/0.5412 | 0.8165/0.9536 |
|  | rs642949 | 536/536 | 0.5053/0.6420 | 0.7219/0.9836 | 464/464 | 0.8691/0.8913 | 1/0.9903 | 238/238 | 0.9772/0.3664 | 1/0.9536 |
| Middle Temporal (volume) | rs592297 | 536/536 | **0.01232**/0.3800 | 0.1232/0.9499 | 464/464 | 0.3774/0.7350 | 1/0.9740 | 238/238 | 0.8482/0.8593 | 0.9425/0.9548 |
|  | rs642949 | 536/536 | **0.02844**/0.2409 | 0.1408/0.8031 | 464/464 | 0.8275/0.6578 | 1/0.9740 | 238/238 | 0.08586/0.3596 | 0.7947/0.5138 |
|  | rs3851179 | 535/535 | 0.06018/0.5146 | 0.1408/1 | 463/463 | 0.5944/0.2663 | 1/0.9740 | 238/238 | 0.4609/0.1109 | 0.7947/0.5071 |
|  | rs561655 | 536/536 | 0.07855/0.8081 | 0.1408/1 | 464/464 | 0.7701/0.4772 | 1/0.9740 | 238/238 | 0.4088/0.2656 | 0.7947/0.5071 |
|  | rs1237999 | 535/535 | 0.09853/0.9235 | 0.1408/1 | 463/463 | 0.9349/0.8259 | 1/0.9740 | 237/237 | 0.5563/0.2566 | 0.7947/0.5071 |
|  | rs7941541 | 536/536 | 0.09754/0.7091 | 0.1408/1 | 464/464 | 0.6797/0.6070 | 1/0.9740 | 238/238 | 0.2836/0.2165 | 0.7947/0.5071 |
|  | rs543293 | 534/534 | 0.08102/0.8752 | 0.1408/1 | 462/462 | 0.9554/0.8766 | 1/0.9740 | 236/236 | 0.3579/0.2552 | 0.7947/0.5071 |
| Parahippocampal (volume) | rs642949 | 536/536 | 0.8764/0.5188 | 1/0.9644 | 464/464 | 0.3753/0.5380 | 0.8903/0.9078 | 237/237 | 0.8046/0.08524 | 1/0.5783 |
|  | rs561655 | 536/536 | 0.5180/0.9962 | 1/0.9644 | 464/464 | 0.7046/0.4277 | 0.8903/0.9078 | 237/237 | 0.9536/0.1851 | 1/0.5783 |
|  | rs7941541 | 536/536 | 0.8869/0.5459 | 1/0.9644 | 464/464 | 0.4145/0.5551 | 0.8903/0.9078 | 237/237 | 0.5709/0.6472 | 1/0.9246 |
|  | rs1237999 | 535/535 | 0.5997/0.6821 | 1/0.9644 | 463/463 | 0.6898/0.3114 | 0.8903/0.9078 | 236/236 | 0.7846/0.2731 | 1/0.5783 |
|  | rs3851179 | 535/535 | 0.6616/0.7715 | 1/0.9644 | 463/463 | 0.5915/0.6355 | 0.8903/0.9078 | 237/237 | 0.5807/0.9156 | 1/1 |
|  | rs592297 | 536/536 | 0.9976/0.5616 | 1/0.9644 | 464/464 | 0.8012/0.8278 | 0.8903/1 | 237/237 | 0.6253/0.2069 | 1/0.5783 |
|  | rs543293 | 534/534 | 0.7999/0.5884 | 1/0.9644 | 462/462 | 0.7102/0.4522 | 0.8903/0.9078 | 235/235 | 0.6712/0.2892 | 1/0.5783 |
| Posterior Cingulate (volume) | rs642949 | 536/536 | 0.1155/**0.01558** | 0.5775/0.0888 | 464/464 | 0.5149/**0.01842** | 0.9189/**0.03069** | 238/238 | 0.3160/0.6334 | 1/0.9048 |
|  | rs3851179 | 535/535 | 0.7952/0.4055 | 1/0.7969 | 463/463 | 0.7371/0.07143 | 0.9189/0.1020 | 238/238 | 0.1438/**0.03199** | 1/0.1106 |
|  | rs7941541 | 536/536 | 0.9454/0.1442 | 1/0.4806 | 464/464 | 0.8270**/0.01765** | 0.9189/**0.03069** | 238/238 | 0.2028/**0.03318** | 1/0.1106 |
|  | rs543293 | 534/534 | 0.7436/0.7569 | 1/1 | 462/462 | 0.8265/**0.009615** | 0.9189/**0.03069** | 236/236 | 0.6036/**0.02317** | 1/0.1106 |
|  | rs561655 | 536/536 | 0.9694/0.4781 | 1/0.7969 | 464/464 | 0.6953/**0.01631** | 0.9189/**0.03069** | 238/238 | 0.4310/0.1255 | 1/0.2511 |
|  | rs592297 | 536/536 | 0.8350/0.3761 | 1/0.7969 | 464/464 | 0.2414/0.2841 | 0.9189/0.3552 | 238/238 | 0.9509/0.7693 | 1/0.9081 |
|  | rs1237999 | 535/535 | 0.7601/0.9359 | 1/1 | 463/463 | 0.7102/**0.003964** | 0.9189/**0.01982** | 237/237 | 0.6371/0.05125 | 1/0.1281 |
| Precuneus (volume) | rs1237999 | 535/535 | 0.1177/0.4509 | 0.3751/1 | 463/463 | 0.2593/0.1772 | 0.4321/0.2954 | 237/237 | 0.9298/0.2911 | 1/0.6148 |
|  | rs543293 | 534/534 | 0.1176/0.5362 | 0.3751/1 | 462/462 | 0.4428/0.2500 | 0.5535/0.3370 | 236/236 | 0.8734/0.3074 | 1/0.6148 |
|  | rs561655 | 536/536 | 0.1500/0.6762 | 0.3751/1 | 464/464 | 0.1668/0.0903 | 0.4169/0.2954 | 238/238 | 0.8241/0.2611 | 1/0.6148 |
|  | rs7941541 | 536/536 | 0.2254/0.8934 | 0.3751/1 | 464/464 | 0.3762/0.1750 | 0.5374/0.2954 | 238/238 | 0.9574/0.5018 | 1/0.6272 |
|  | rs642949 | 536/536 | 0.2248/0.0533 | 0.3751/0.2576 | 464/464 | 0.2135/**0.04854** | 0.4270/0.2954 | 238/238 | 0.3741/0.09117 | 1/0.4940 |
|  | rs3851179 | 535/535 | 0.3082/0.9576 | 0.4402/1 | 463/463 | 0.09416/0.1759 | 0.3139/0.2954 | 238/238 | 0.8428/0.6618 | 1/0.7353 |
|  | rs592297 | 536/536 | 0.5305/0.9562 | 0.5894/1 | 464/464 | 0.8778/0.5596 | 0.9753/0.6217 | 238/238 | 0.9322/0.09881 | 1/0.4940 |
| CA1 region (volume) | rs1237999 | 535/535 | 0.7461/0.9628 | 0.9120/1 | 463/463 | 0.2036/**0.03896** | 0.5794/0.1948 | 236/236 | 0.8315/0.8147 | 1/1 |
|  | rs3851179 | 535/535 | 0.6220/0.6215 | 0.9120/1 | 463/463 | 0.4948/0.3608 | 0.6185/0.6455 | 237/237 | 0.8292/0.9705 | 1/1 |
|  | rs543293 | 534/534 | 0.8208/0.7907 | 0.9120/1 | 462/462 | 0.1770/**0.03102** | 0.5794/0.1948 | 235/235 | 0.9298/0.9983 | 1/1 |
|  | rs561655 | 536/536 | 0.6230/0.7800 | 0.9120/1 | 464/464 | 0.3466/0.2208 | 0.6185/0.5519 | 237/237 | 0.9366/0.8043 | 1/1 |
|  | rs592297 | 536/536 | 0.3637/0.9818 | 0.9120/1 | 464/464 | 0.1822/0.6662 | 0.5794/0.7402 | 237/237 | 0.6562/0.9505 | 1/1 |
|  | rs642949 | 536/536 | 0.3648/0.5087 | 0.9120/1 | 464/464 | 0.2317/0.6100 | 0.5794/0.7402 | 237/237 | 0.7175/0.5968 | 1/1 |
|  | rs7941541 | 536/536 | 0.7680/0.9446 | 0.9120/1 | 464/464 | 0.4746/0.09777 | 0.6185/0.3259 | 237/237 | 0.9279/0.7599 | 1/1 |
| Hippocampus (volume) | rs592297 | 536/536 | 0.3604/0.6912 | 1/0.9185 | 464/464 | 0.9634/0.9180 | 1/1 | 238/238 | 0.7884/0.5806 | 1/1 |
|  | rs642949 | 536/536 | 0.5503/0.7180 | 1/0.9185 | 464/464 | **0.04012**/0.6456 | 0.4012/1 | 238/238 | 0.9046/0.3254 | 1/1 |
|  | rs561655 | 536/536 | 0.7327/0.5330 | 1/0.9185 | 464/464 | 0.8069/0.9364 | 1/1 | 238/238 | 0.9027/0.6852 | 1/1 |
|  | rs1237999 | 535/535 | 0.9098/0.7348 | 1/0.9185 | 463/463 | 0.6733/0.9182 | 1/1 | 237/237 | 0.9989/0.6884 | 1/1 |
|  | rs543293 | 534/534 | 0.9352/0.8328 | 1/0.9253 | 462/462 | 0.4851/0.4286 | 1/1 | 236/236 | 0.9431/0.9258 | 1/1 |
|  | rs7941541 | 536/536 | 0.8800/0.7110 | 1/0.9185 | 464/464 | 0.8611/0.5229 | 1/1 | 238/238 | 0.9806/0.9670 | 1/1 |
|  | rs3851179 | 535/535 | 0.8517/0.6276 | 1/0.9185 | 463/463 | 0.8203/0.9956 | 0.9457/0.9993 | 238/238 | 0.8707/0.8791 | 1/1 |
| Entorhinal (thickness) | rs592297 | 536/536 | 0.4236/0.9527 | 0.8922/1 | 464/464 | 0.6002/0.8525 | 0.9756/1 | 238/238 | 0.6687/0.9815 | 0.8504/1 |
|  | rs642949 | 536/536 | 0.6245/0.7019 | 0.8922/1 | 464/464 | 0.6829/0.2062 | 0.9756/1 | 238/238 | 0.4277/0.8209 | 0.8504/1 |
|  | rs561655 | 536/536 | 0.8705/0.2136 | 0.9849/0.6604 | 464/464 | 0.1459/0.6182 | 0.5495/1 | 238/238 | 0.4456/0.5504 | 0.8504/1 |
|  | rs1237999 | 535/535 | 0.5621/0.4515 | 0.8922/0.8557 | 463/463 | 0.1898/0.8362 | 0.5495/1 | 237/237 | 0.6803/0.2637 | 0.8504/0.8789 |
|  | rs543293 | 534/534 | 0.5436/0.5134 | 0.8922/0.8557 | 462/462 | 0.2802/0.9701 | 0.5604/1 | 236/236 | 0.6716/0.2458 | 0.8504/0.8789 |
|  | rs7941541 | 536/536 | 0.8864/0.2642 | 0.9849/0.6604 | 464/464 | 0.2198/0.9726 | 0.5495/1 | 238/238 | 0.5234/0.3607 | 0.8504/0.9018 |
|  | rs3851179 | 535/535 | 0.5664/**0.04615** | 0.8922/0.4615 | 463/463 | 0.1576/0.4564 | 0.5495/1 | 238/238 | 0.2105/0.7788 | 0.8504/1 |
| Middle Temporal (thickness) | rs592297 | 536/536 | 0.1202/0.9381 | 0.6011 /1 | 464/464 | 0.9735/0.1963 | 1/0.4939 | 238/238 | 0.6082/0.2880 | 0.9281/0.8094 |
|  | rs642949 | 536/536 | 0.1897/0.2172 | 0.6323 /0.7240 | 464/464 | 0.1456/0.5315 | 0.7179/0.5905 | 238/238 | **0.01574**/**0.0420** | 0.1574/0.4200 |
|  | rs561655 | 536/536 | 0.4286/0.4201 | 0.6529 /0.8898 | 464/464 | 0.8546/0.1451 | 1/0.4939 | 238/238 | 0.4153/0.4336 | 0.8307/0.8671 |
|  | rs1237999 | 535/535 | 0.3831/0.6950 | 0.6529 /0.9929 | 463/463 | 0.8784/0.3156 | 1/0.5261 | 237/237 | 0.8353/0.5801 | 0.9281/0.9011 |
|  | rs543293 | 534/534 | 0.3581/0.6525 | 0.6529 /0.9929 | 462/462 | 0.8519/0.3896 | 1/0.5565 | 236/236 | 0.6497/0.6703 | 0.9281/0.9011 |
|  | rs7941541 | 536/536 | 0.4570/0.4449 | 0.6529 /0.8898 | 464/464 | 0.9684/0.1975 | 1/0.4939 | 238/238 | 0.3336/0.7209 | 0.8307/0.9011 |
|  | rs3851179 | 535/535 | 0.5862/0.2007 | 0.7328 /0.7240 | 463/463 | 0.9853/0.1227 | 1/0.4939 | 238/238 | 0.3756/0.9971 | 0.8307/1 |
| Parahippocampal (thickness) | rs592297 | 536/536 | **0.03263**/0.1293 | 0.1088/0.2599 | 464/464 | 0.6106/0.4595 | 0.9819/0.6160 | 238/238 | 0.5111/0.8737 | 0.9403/1 |
|  | rs642949 | 536/536 | **0.02702**/0.1563 | 0.1088/0.2599 | 464/464 | 0.1537/0.3443 | 0.9819/0.6160 | 238/238 | 0.08819/**0.04542** | 0.8819/0.4542 |
|  | rs561655 | 536/536 | **0.01473**/**0.03267** | 0.1088/0.2599 | 464/464 | 0.6873/0.1470 | 0.9819/0.6160 | 238/238 | 0.8462/0.7389 | 0.9403/1 |
|  | rs1237999 | 535/535 | 0.06434/0.1093 | 0.1390/0.2599 | 463/463 | 0.9874/0.2553 | 0.9819/0.6160 | 237/237 | 0.6941/0.9707 | 0.9403/1 |
|  | rs543293 | 534/534 | 0.08339/0.1806 | 0.1390/0.2599 | 462/462 | 0.5864/0.3880 | 0.9819/0.6160 | 236/236 | 0.7529/0.9027 | 0.9403/1 |
|  | rs7941541 | 536/536 | 0.1354/0.2641 | 0.1934/0.2934 | 464/464 | 0.5174/0.4928 | 0.9819/0.6160 | 238/238 | 0.8238/0.9417 | 0.9403/1 |
|  | rs3851179 | 535/535 | 0.06977/0.07096 | 0.1390/0.2599 | 463/463 | 0.4733/0.1645 | 0.9819/0.6160 | 238/238 | 0.8366/0.4888 | 0.9403/1 |
| Posterior Cingulate (thickness) | rs592297 | 536/536 | 0.1194/0.6806 | 1 /0.9463 | 464/464 | 0.2451/**0.002504** | 1/**0.02504** | 238/238 | 0.7444/0.7124 | 0.8272/1 |
|  | rs642949 | 536/536 | 0.2230/0.2305 | 1 /0.9463 | 464/464 | 0.4112/0.9115 | 1/1 | 238/238 | 0.3977/0.8476 | 0.8272/1 |
|  | rs561655 | 536/536 | 0.5569/0.6686 | 1 /0.9463 | 464/464 | 0.7728/0.3378 | 1/0.6803 | 238/238 | 0.4927/0.9830 | 0.8272/1 |
|  | rs1237999 | 535/535 | 0.8036/0.5848 | 1 /0.9463 | 463/463 | 0.5955/0.2224 | 1/0.6803 | 237/237 | 0.7442/0.7362 | 0.8272/1 |
|  | rs543293 | 534/534 | 0.9583/0.6160 | 1 /0.9463 | 462/462 | 0.9249/0.3402 | 1/0.6803 | 236/236 | 0.6828/0.7719 | 0.8272/1 |
|  | rs7941541 | 536/536 | 0.9442/0.8517 | 1 /0.9463 | 464/464 | 0.8517/0.5292 | 1/0.6973 | 238/238 | 0.3344/0.6610 | 0.8272/1 |
|  | rs3851179 | 535/535 | 0.9767/0.7738 | 1 /0.9463 | 463/463 | 0.7128/0.5579 | 1/0.6973 | 238/238 | 0.6404/0.6951 | 0.8272/1 |
| Precuneus (thickness) | rs592297 | 536/536 | 0.5007/0.6468 | 1 /0.7187 | 464/464 | 0.8975/0.7152 | 1/1 | 238/238 | 0.2778/0.4402 | 1 /0.4891 |
|  | rs642949 | 536/536 | **0.03506**/0.3518 | 0.3506 /0.5463 | 464/464 | 0.8042/0.9872 | 1/1 | 238/238 | **0.04728**/0.2226 | 0.6151/0.3180 |
|  | rs561655 | 536/536 | 0.8277/0.3136 | 1 /0.5463 | 464/464 | 0.8955/0.9245 | 1/1 | 238/238 | **0.02804**/0.1178 | 0.6151/0.3180 |
|  | rs1237999 | 535/535 | 0.6212/0.5057 | 1 /0.6322 | 463/463 | 0.8869/0.6652 | 1/1 | 237/237 | 0.0840/0.1632 | 0.6151/0.3180 |
|  | rs543293 | 534/534 | 0.7518/0.3492 | 1 /0.5463 | 462/462 | 0.7140/0.5986 | 1/1 | 238/238 | 0.1108/0.1664 | 0.6181/0.3180 |
|  | rs7941541 | 536/536 | 0.9792/0.2399 | 1 /0.5463 | 464/464 | 0.7508/0.8678 | 1/1 | 238/238 | 0.1266/0.2588 | 0.6181/0.3235 |
|  | rs3851179 | 535/535 | 0.5045/0.05789 | 1 /0.5463 | 463/463 | 0.9143/0.9769 | 1/1 | 238/238 | 0.07941/0.1456 | 0.6151/0.3180 |

R=right; L= left; Pc= p value after controlling for confounders

**Supplementary table 3A- Validation results of relationship between *PICALM* variants and volume/thickness of posterior cingulate in subgroup population.**

| **Phenotype** | **Subgroup** | **Seven SNPs selected for analysis** | | | | | | |
| --- | --- | --- | --- | --- | --- | --- | --- | --- |
|  |  | **rs3851179** | **rs561655** | **rs543293** | **rs592297** | **rs7941541** | **rs1237999** | **rs642949** |
| **Left/Right Posterior cingulate** | **NC** | ● | ●**☆** | **☆☆** | **☆** | ● | **☆** | ★ |
|  | **MCI** | ∕ | ∕ | ● | **☆** | ∕ | ∕ | ∕ |

★ Baseline; **☆** 1 year of follow-up; ● 2 year of follow-up;

**Abbreviation**: SNP= Single Nucleotide Polymorphism

**Supplementary table 3B Detailed results of the associations of *PICALM* loci with volume/thickness of posterior cingulate in subgroup population**

| **Subgroup** | **Phenotypes** | **SNP** | **Baseline** | | | **Follow-up (1 year)** | | | **Follow-up (2 year)** | | |
| --- | --- | --- | --- | --- | --- | --- | --- | --- | --- | --- | --- |
|  |  |  | **Sample (L/R)** | **P-value (L/R)** | **Sample (L/R)** | | **P-value (L/R)** | **Sample (L/R)** | | **P-value (L/R)** |  |
| NC | Volume | rs642949 | 159/159 | 0.07583/0.4351 | 139/139 | | 0.6206/0.2122 | 60/60 | | 0.8179/0.7712 |  |
|  |  | rs3851179 | 158/158 | 0.4642/0.6170 | 138/138 | | 0.9831/0.1436 | 60/60 | | **0.002711**/0.8971 |  |
|  |  | rs7941541 | 159/159 | 0.3183/0.2234 | 139/139 | | 0.7554/0.1079 | 60/60 | | **0.01834**/0.9518 |  |
|  |  | rs543293 | 157/157 | 0.5048/0.6228 | 137/137 | | 0.4684/**0.008868** | 60/60 | | 0.2202/0.9448 |  |
|  |  | rs561655 | 159/159 | 0.1891/0.2290 | 139/139 | | 0.5664/**0.02829** | 60/60 | | **0.02297**/0.7894 |  |
|  |  | rs592297 | 159/159 | 0.2998/0.2005 | 139/139 | | 0.7630/0.1957 | 60/60 | | 0.1163/0.1527 |  |
|  |  | rs1237999 | 158/158 | 0.5950/0.8366 | 138/138 | | 0.6122/**0.003204** | 60/60 | | 0.3036/0.7853 |  |
|  | Thickness | rs592297 | 159/159 | 0.4484/0.2403 | 139/139 | | 0.3388/**0.006687** | 60/60 | | 0.7407/0.8791 |  |
|  |  | rs642949 | 159/159 | **0.01761**/0.2397 | 139/139 | | 0.2729/0.1202 | 60/60 | | 0.3569/0.7641 |  |
|  |  | rs561655 | 159/159 | 0.6572/0.1258 | 139/139 | | 0.06558/0.1028 | 60/60 | | 0.1673/0.2416 |  |
|  |  | rs1237999 | 158/158 | 0.9080/0.2694 | 139/139 | | 0.1342/0.06798 | 59/59 | | 0.3604/0.3036 |  |
|  |  | rs543293 | 157/157 | 0.8544/0.2451 | 137/137 | | 0.1470/**0.0450** | 58/58 | | 0.2649/0.2515 |  |
|  |  | rs7941541 | 159/159 | 0.6851/0.3444 | 139/139 | | 0.2638/0.3077 | 60/60 | | 0.1053/0.1693 |  |
|  |  | rs3851179 | 158/158 | 0.6872/0.5274 | 138/138 | | 0.5948/0.2736 | 60/60 | | 0.2847/0.09377 |  |
| MCI | Volume | rs642949 | 332/332 | 0.2435/0.07648 | 295/295 | | 0.5525/0.1110 | 168/168 | | 0.2683/0.5875 |  |
|  |  | rs3851179 | 332/332 | 0.8495/0.9187 | 295/295 | | 0.7099/0.3617 | 168/168 | | 0.6685/0.07288 |  |
|  |  | rs7941541 | 332/332 | 0.6239/0.5721 | 295/295 | | 0.3122/0.1287 | 168/168 | | 0.9730/0.0563 |  |
|  |  | rs543293 | 332/332 | 0.3791/0.8314 | 295/295 | | 0.1522/0.2266 | 168/168 | | 0.7186/**0.03826** |  |
|  |  | rs561655 | 332/332 | 0.4010/0.8012 | 295/295 | | 0.4907/0.2318 | 168/168 | | 0.7056/0.2022 |  |
|  |  | rs592297 | 332/332 | 0.2197/0.9256 | 295/295 | | 0.1413/0.1864 | 168/168 | | 0.6427/0.5435 |  |
|  |  | rs1237999 | 332/332 | 0.4267/0.6307 | 295/295 | | 0.4204/0.1335 | 168/168 | | 0.8056/0.0606 |  |
|  | Thickness | rs592297 | 332/332 | 0.3877/0.2315 | 295/295 | | 0.2364/**0.02215** | 168/168 | | 0.9450/0.7365 |  |
|  |  | rs642949 | 332/332 | 0.6339/0.4912 | 295/295 | | 0.6167/0.9392 | 168/168 | | 0.9628/0.8712 |  |
|  |  | rs561655 | 332/332 | 0.7692/0.6054 | 295/295 | | 0.9984/0.5384 | 168/168 | | 0.7390/0.3604 |  |
|  |  | rs1237999 | 332/332 | 0.7884/0.7103 | 295/295 | | 0.9612/0.5425 | 168/168 | | 0.5367/0.5343 |  |
|  |  | rs543293 | 332/332 | 0.9004/0.6864 | 295/295 | | 0.5291/0.8448 | 168/168 | | 0.4635/0.4874 |  |
|  |  | rs7941541 | 332/332 | 0.9690/0.6202 | 295/295 | | 0.6184/0.6361 | 168/168 | | 0.7569/0.5571 |  |
|  |  | rs3851179 | 332/332 | 0.9573/0.3728 | 295/295 | | 0.9336/0.7609 | 168/168 | | 0.6687/0.09687 |  |
